# Simple and cost-effective laboratory methods to evaluate and validate cell-free DNA isolation

**DOI:** 10.1186/s13104-018-3866-8

**Published:** 2018-10-23

**Authors:** Afsaneh Mojtabanezhad Shariatpanahi, Parisa Rokni, Elaheh Shahabi, Fatemeh Varshoee Tabrizi, Mohammad Amin Kerachian

**Affiliations:** 1 Cancer Genetics Research Unit, Reza Radiotherapy and Oncology Center, Mashhad, Iran; 2 Department of Medical Genetic, Tabriz University of Medical Sciences, Tabriz, Iran; 3 Department of Biology, Faculty of Science, University of Guilan, Rasht, Iran; 4 Medical Genetics Research Center, Mashhad University of Medical Sciences, Mashhad, Iran; 5 Department of Medical Genetics, Faculty of Medicine, Mashhad University of Medical Sciences, Mashhad, Iran

**Keywords:** Plasma, Liquid biopsy, Cell-free DNA

## Abstract

**Objective:**

In the present study, we investigated different simple and cost effective methods to evaluate and validate cell free DNA (cfDNA) isolation. The ability of the QIAamp DNA Blood Mini Kit method to extract cfDNA was assessed by several approaches, including purification of endogenous cfDNA and exogenous spike-in control material, prior to plasma extraction, and followed by quantitative-PCR.

**Results:**

Using QIAamp DNA Blood Mini kit, nearly 27% (380 bp) to 35% (173 bp) cfDNA was recovered with a higher recovery of smaller size cfDNA (173 bp) in comparison to larger ones (380 bp). These simple laboratory methods can be used to assess the efficiency of any cfDNA isolation method.

**Electronic supplementary material:**

The online version of this article (10.1186/s13104-018-3866-8) contains supplementary material, which is available to authorized users.

## Introduction

Circulating cell-free DNA (cfDNA) molecules are shed into bloodstream, plasma, serum, urine as well as other body fluids of humans [1]. Most evidence suggests that the released cfDNA is primarily a consequence of apoptosis that could be released by various pathologic and normal physiologic mechanisms [2]. Evaluation and quantification of cfDNA in plasma or serum, termed “liquid biopsy” has become one of the most important clinical analysis for early cancer detection, genetic and epigenetic monitoring, recurrence prediction, therapeutic resistance assessment of cancer and prenatal testing [1–3]. Comprehensive liquid biopsy analysis may also represent a tool to assess both tumor burden and molecular features of disease [4]. Despite the extensive clinical utility of cfDNA, there are some challenges and difficulties in its extraction and subsequently its usage for example cfDNA is mostly fragmented and exists at very low concentrations in plasma. Isolation, quantification and evaluation of cfDNA is not a straightforward task and it requires a sensitive and reliable workflow. Therefore, the present study designed and performed different simple experiments to isolate cfDNA and validate the extraction.

## Main text

### Sample collection and preparation

Plasma samples were chosen for evaluation of cfDNA extraction since the extracted cfDNA from plasma contains a lower background concentration of wild-type DNA in comparison to serum [2]. Peripheral blood was collected from healthy volunteers, placed into EDTA tubes and kept at room temperature (18 °C–22 °C) for no more than 2 h. Thereafter, the blood sample was centrifuged twice at 800*g* for 10 min followed by 1600*g* for 10 min. The plasma was separated and transferred to another tube and stored at − 80 °C for further analysis.

### Circulating cell-free DNA extraction

Plasma samples were thawed prior to Qiagen extraction. In accordance with the manufacturer’s instructions, DNA was purified using the QIAamp Blood DNA Mini Kit (Qiagen, UK) by fast spin-column procedures. The protocol was performed by using 300 µl plasma and eluting the DNA with 30 µl of elution buffer (AE) in 2 steps, with 20 µl and 10 µl AE and incubation time of 5 min each.

### Experiment 1: following the spiked 100 base-pair DNA ladder

To evaluate the bias associated with fragment size, and to determine if the DNA extraction method has led to loss of small or large DNA fragments, the following approach was used. Plasma of a healthy human was prepared as previously described. Ten microliter of GeneRuler 100 bp (Thermo-Scientific, United States) as a cfDNA was spiked in 300 μl plasma sample. The extraction was performed and the purified DNAs were analyzed on a 2% UltraPure Agarose gel (Invitrogen, United States) stained by Green Viewer (Parstous Biotechnology Co., Iran).

### Experiment 2: looking for Y-chromosome-specific sequences in male-bearing pregnancy

To ensure the presence of cfDNA in the extracted DNA, Y-chromosome-specific sequence (*DYS221* locus), which represents male fetuses and does not exist in the maternal genome, was sought in male-bearing pregnancy in the maternal blood. Cell-free fetal DNA fraction represents approximately 3–13% of the total cell-free maternal DNA plasma. Since a median of 99% of the fetal-derived DNA molecules was less than 313 base pair (bp) in length [5], a 173 bp amplicon of *DYS221* locus was considered for amplification.

One and a half milliliter of plasma obtained from a woman with male-bearing pregnancy was prepared. Pooled plasma was divided into 5 micro-tubes (300 µl in each), and then extractions were performed. All these samples were evaluated by SYBR-Green real-time PCR in triplicate. DNA plasmas from a non-pregnant female and a male were considered as negative and positive controls, respectively. The quantitative PCR (qPCR) assays were performed according to the MIQE guidelines.

PCR amplifications were carried out on a LightCycler^®^ 96 System (Roche, Germany). PCR was carried out in a 15 µl total volume using HiFi SYBR Green Master Mix (Farabin, Iran), 300 nM of each primer (Additional file 1: Table S1) , and 2.5 µl of DNA template. The amplification was consisted of 15 min at 95 °C, followed by 50 cycles of 20 s at 95 °C, 15 s at the primer annealing temperature of 59 °C and 15 s at 72 °C.

### Experiment 3: following spiked bisulfite DNA

To assess the extraction efficiency, pooled plasma of a healthy human was divided into five micro-tubes (300 µl in each). EpiTect Control Unmethylated Bisulfite DNA (Qiagen, Germany) was spiked into all the plasmas, at a final concentration of 75 ng/ml. Spiked bisulfite DNA was re-purified using QIAamp Blood DNA Mini kit extraction method.

Each sample was evaluated by SYBR-Green real-time PCR with bisulfite specific PCR (BSP) primers for *Bone Morphogenic Protein 3* gene (*BMP*_*3*_, 256 bp) [6] in triplicate. BSP primers specifically amplify bisulfite converted DNA (Additional file 1: Table S1). Genomic DNA was used in qPCR as a negative control and bisulfite unmethylated DNA as a positive control. The qPCR assays were performed according to the MIQE guidelines.

Since a large fraction of DNA will be lost during bisulfite conversion, SYBR-Green nested qPCR assay was carried out [7]. PCR amplifications were run on a LightCycler^®^ 96 System (Roche, Germany). PCR was carried out in a 15 µl total volume using HiFi SYBR Green Master Mix (Farabin, Iran), 300 nM of each primer, 0.2 µg/µl bovine serum albumin and 2.5 µl of bisulfite-modified template. The amplification was consisted of 15 min at 95 °C, followed by 15 cycles of 20 s at 95 °C, 15 s at the primer annealing temperature of 60 °C and 15 s at 72 °C.

For the second round of amplification, 2 μl of the PCR product from the initial amplification was used as the template. The condition of second round qPCR was 15 min at 95 °C, followed by 40 cycles of 20 s at 95 °C, 15 s at the primer annealing temperature of 60 °C and 15 s at 72 °C.

### Experiment 4: following spiked male genomic DNA

To measure the absolute quantification of cfDNA extracted by Qiagen method, pooled plasma of healthy woman was divided in 11 microtubes (300 µl in each). Fragmented male genomic blood DNA was spiked at 1600 ng/ml (1.6 ng/µl) as final concentration in each plasma (10 microtubes). One remaining unspiked microtube was used as a negative control. To assess the recovery of the exogenous spike-in from cfDNA extraction, a seven-point two-fold dilution series (from 0.25 ng/µl to 16 ng/µl) of the genomic DNA from male blood (diluent, nuclease-free water) was used to draw standard curve for Y-chromosome-specific sequences: *DYS221* locus (173 bp) and *DAZ* locus (380 bp) separately (Additional file 1: Table S1).

All samples were evaluated by SYBR-Green qPCR in triplicate and the mean value was used for quantification. Its efficiency was calculated based on the slope of the standard curve (equation: efficiency = (10^(−1/slope)^ − 1) × 100) and all correlation coefficients (r^2^) ≥ 0.99 were considered. Melting curves of all samples were observed carefully to ensure that only one product was amplified. The qPCR assays were performed according to the *MIQE* guidelines.

### Experiment 5: Kras mutation monitoring

To show the presence of cell-free tumor DNA in cfDNA extractions, *Kras* mutation analysis was performed on the extracted cfDNA from plasma of a *Kras* positive colorectal cancer patient (exon 2, codons 12, G12D mutation).

Mutation detection was performed with a clinical validated approach based on the allele-specific qPCR intplex method [8]. PCR cycling analysis was conducted on LightCycler^®^96 Real-Time PCR System.

Design of qPCR primer systems were such that it could amplify two amplicons within a region of the target mutant: one corresponding to a mutated allele specific amplicon (61 bp, melting temperature about 81 °C) and the other to a wild-type reference amplicon (67 bp, melting temperature: about 75 °C). The test discriminates highly and specifically between mutant and wild-type alleles using a blocking 3′-phosphate modified oligonucleotide and low Tm primers.

The PCR conditions for *KRAS* were as follows: each 15 µl-triplicate reaction contained 2 µl of purified DNA diluted in 5X HOT FIREPol^®^ EvaGreen^®^ qPCR Mix Plus HRM Master Mix and 200 nM primers (Additional file 1: Table S1), with the following PCR program and melting conditions used for all amplicons: 95 °C for 15 min; 45 cycles of 95 °C for 15 s and 60 °C for 20 s; 90 °C for 30 s, followed by a high resolution melt of 55–90 °C (0.01 °C/s, 45 acquisitions/°C). Data were acquired and analyzed using the accompanying High Resolution Melt software 1.1v. All HRM reactions were run in triplicate.

Quantitative results were measured as mean ± standard deviation (SD) and differences were assessed by two-tailed unpaired t-test. The reproducibility of different protocols was evaluated by coefficient of variation (CV). p-value less than 0.05 was considered statistically significant.

### Experiment 1: result

**Fig. 1:**
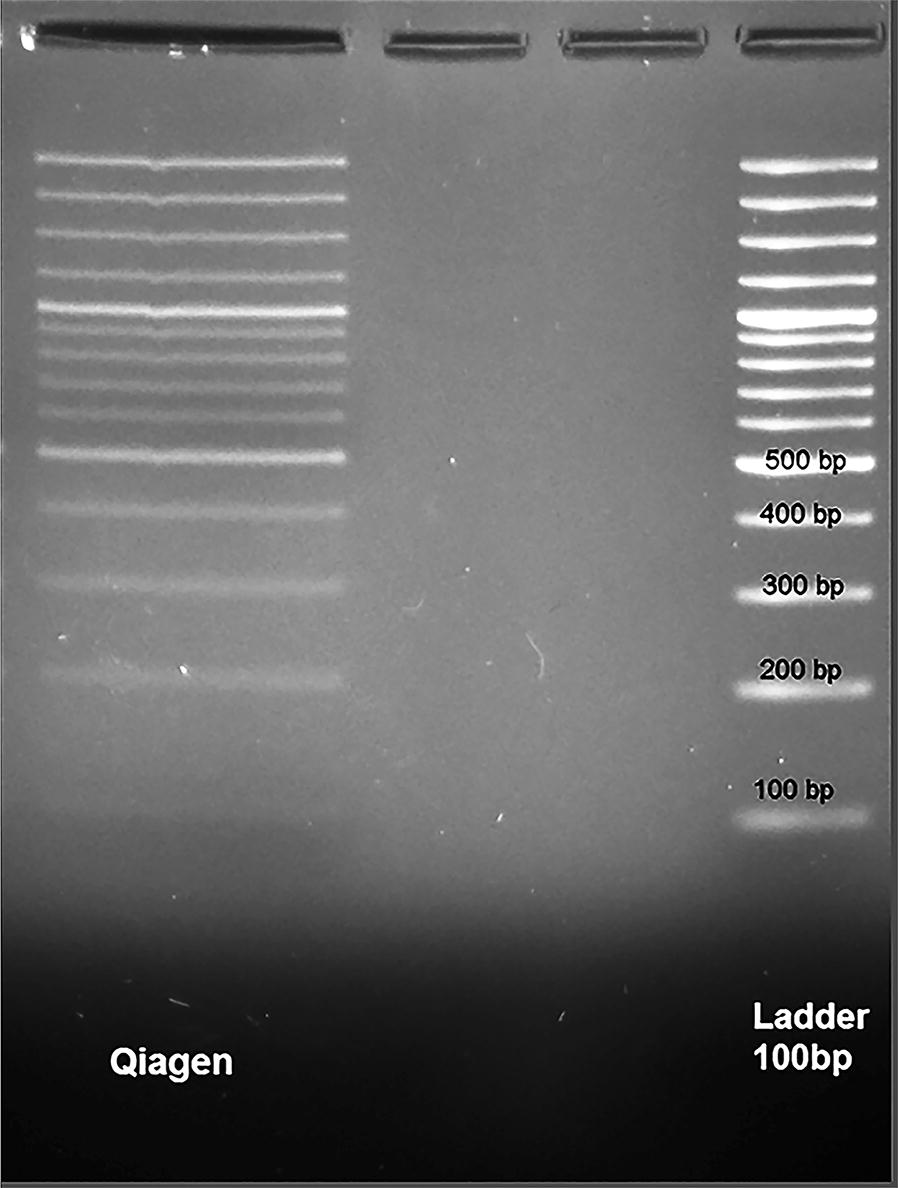
The purified cfDNA analysis on a 2% agarose gel

After re-purification, the DNA ladder was analyzed on 2% UltraPure Agarose gels by Green Viewer as shown in Fig. 1. The figure displays that all sizes of DNA ladder were purified with Qiagen method similar to the ladder configuration.

### Experiment 2: result

SYBR-Green qPCR were performed in triplicate for *DYS221* locus (173 bp). Melting curves of all samples were observed for every reaction to ensure that only one product was amplified. Three out of 5 Qiagen (Cq mean: 35.1) samples were successfully amplified indicating the presence of cfDNA in extracted DNA in this method (Additional file 2: Fig. S1).

### Experiment 3: result

SYBR-Green nested qPCR were performed in triplicate for *BMP*_*3*_ (256 bp). Results showed that all Qiagen (Cq mean: 33.3) samples were successfully amplified by the SYBR-Green nested qPCR (Additional file 3: Fig. S2).

### Experiment 4: result

CfDNA extraction for Qiagen method were performed in 10 replicates and each sample was quantified by SYBR-Green qPCR in triplicate for *DAZ* locus (380 bp) and *DYS221* locus (173 bp) separately.

The standard curve of the *DAZ* and *DYS221* amplicon showed a PCR efficiency of 100% and 94% respectively. The linear regression analysis of mean cycle threshold values per triplicate against log concentrations in the dilution yielded R^2^ = 1 for *DAZ* and R^2^ = 0.99 for *DYS221* locus (Additional file 4: Fig. S3).

**Fig. 2:**
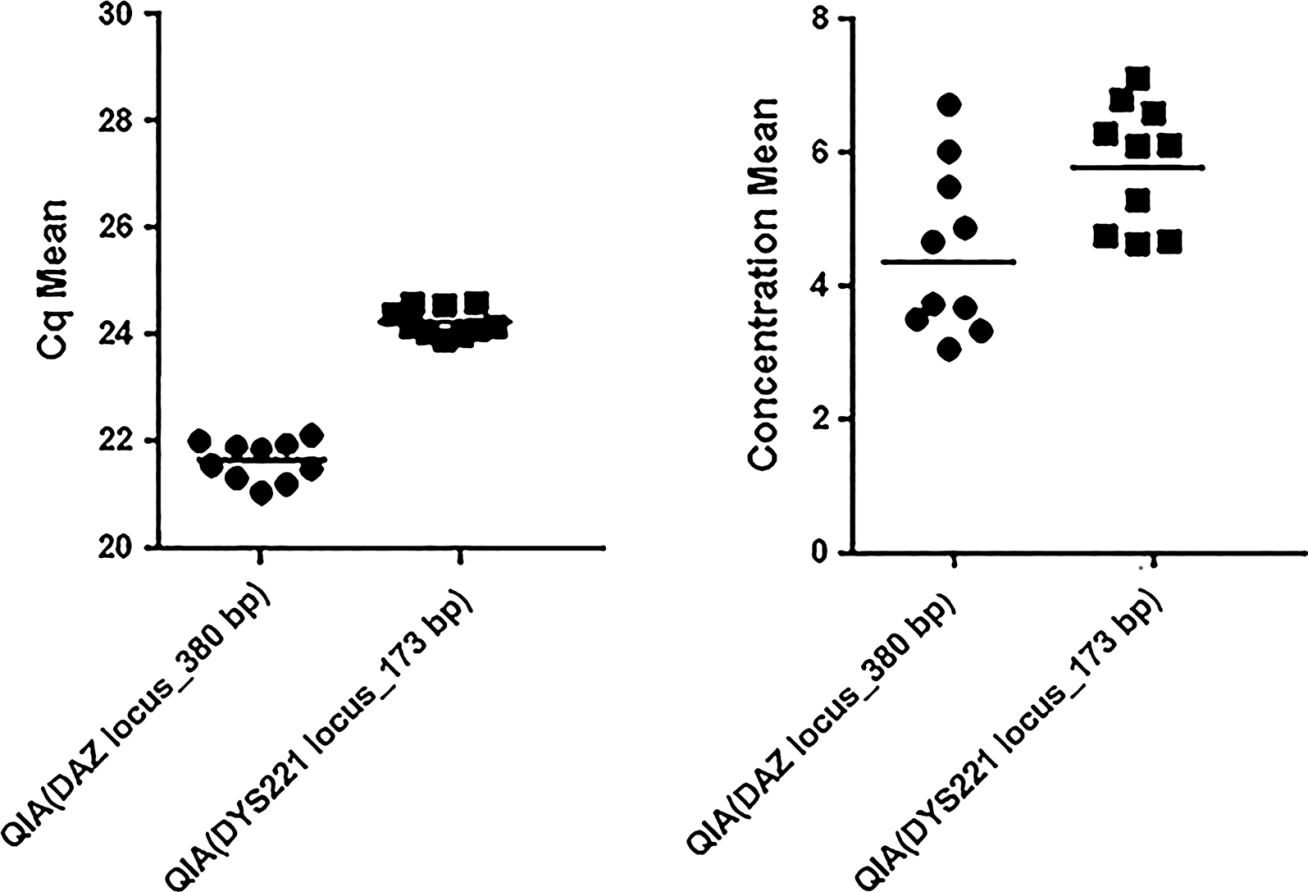
Cq mean and concentration mean (ng/µl)

All Qiagen samples were successfully amplified by the SYBR-Green qPCR assay. The mean ± SD of Cq-value was 21.6 ± 0.37 (range 21.0–22.1) for *DAZ* locus and 24.2 ± 0.26 (range 23.9–24.6) for *DYS221* locus. The mean ± SD of concentration value was 4.54 ± 1.25 (range 3.1–6.75) for *DAZ* locus and 5.85 ± 0.93 (range: 4.66–7.14) for *DYS221* locus. Data were presented as scatter dot plot (Fig. 2). The Cq coefficients of variation (CV) was 1.74% for *DAZ* and 1.07% for *DYS221* locus and the concentration CV was 27.56% and 15.89% for *DAZ* and *DYS221* locus, respectively.

Results showed that the recovery of Qiagen cfDNA extraction was about 27% for *DAZ* locus and 35% for *DYS221* locus and this kit had a CV of 15%, 26%, for *DAZ* and *DYS221* loci, respectively.

### Experiment 5: result

**Fig. 3:**
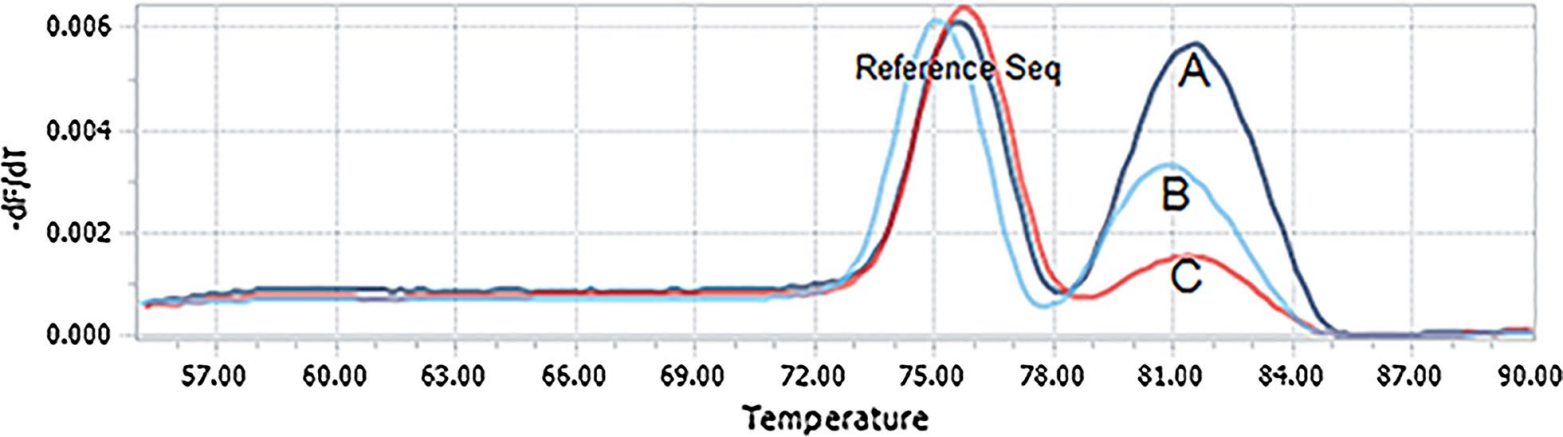
Schematics of the methodology and the melting curve analysis of Kras (35G > A; G12D) point mutation detection (malting temperature: 81 °C). **a** Tissue of *Kras* positive colorectal cancer patient. **b**, **c** Plasma cfDNA extraction by Qiagen (duplicate)

Results indicated that *Kras* mutation was detected in cfDNA extracted by Qiagen method in plasma of a colorectal cancer patient whose colon biopsy was already *Kras* positive (Fig. 3).

Sample handling and the techniques used for cfDNA analysis are one of the major hindrances in cfDNA studies [9, 10]. Pre-analytical factors i.e. every step from blood draw to sample collection and storage of DNA, potentially affecting cfDNA concentration and fragmentation, should be taken into account during data analysis [9, 11].

We demonstrated that using Qiagen kit, nearly 27% (*DAZ*, 380 bp) to 35% (*DYS221*, 173 bp) cfDNA was recovered with a higher recovery for smaller size cfDNA (*DYS221*, 173 bp) in comparison to larger ones (*DAZ*, 380 bp). Xue et al. reported the efficiency of DNA extraction was 18.6% using the standard QIAamp Blood mini kit [12]. Fleischhacker et al. showed the median values for the quantitation for QIAamp DNA Blood Midi Kit was about 1.6–2.7 ng/ml [13].

## Limitation

This study has some limitations. Studies with larger sample size, could have more robust and consistent results. The results of some experiments could be affected due to the very fragmented and low concentration of cfDNA in plasma. The cfDNA extracted from plasma is instable and could be degraded by the passage of time as experienced by many scientists.

## Additional files

**Additional file 1: Table S1.** Primer sequences.

**Additional file 2: Fig. S1.** SYBR Green real-time PCR for DYS221 locus in male bearing pregnancy. (a) Amplification Curve. (b) Melting peak.

**Additional file 3: Fig. S2.** Amplification curve of BMP3 gene in following spiked bisulfite DNA experiment.

**Additional file 4: Fig. S3.** The standard curve of the DAZ and DYS221 amplicon.

## Abbreviations

cfDNA: cell free DNA
bp: base pair
AE: elution buffer
qPCR: quantitative polymerase chain reactions
BSP: bisulfite specific polymerase chain reactions
SD: standard deviation
CV: coefficient of variation
