# Supplementary material for: Simple and cost-effective laboratory methods to evaluate and validate cell-free DNA isolation
Source: BMC Res Notes. 2018 Oct 23;11:757. doi: 10.1186/s13104-018-3866-8 (PMC6199704; doi:10.1186/s13104-018-3866-8)
Supplement: Supplementary file 1 — Additional file 1: Table S1. Primer sequences. [file 13104_2018_3866_MOESM1_ESM.docx]

| Blocker  5^’^ > 3’ | Reverse Primer  5^’^ > 3^’^ | Forward Primer  5^’^ > 3^’^ | Locus |
| --- | --- | --- | --- |
| - | TGGGAATCACTTTTGCAACT | AGAGAGTTTTCTAACAGGGCG | **DYS221** |
| - | GAACCGTATCTACCAAAGCAGC | GGGTGTTACCAGAAGGCAAA | **DAZ** |
| - | AATTAAACTCCAAACCAACTAAAAC | GAGGAGGGAAGGTATAGATAGA | **BMP_3_** |
| GCCTACGCCACCAGCTC - PHO | CTCTTGCCTACGCCAT | GCCTGCTGAAAATGACTGA | **Kras mutant**  **(35G>A; G12D)** |
| - | CCCTGACATACTCCCAAGGA | CCTTGGGTTTCAAGTTATATG | **Kras**  **(Ref Seq)** |

*S1 Table. Primer sequences.*
