# Supplementary figures and images for: Simple and cost-effective laboratory methods to evaluate and validate cell-free DNA isolation
Source: BMC Res Notes. 2018 Oct 23;11:757. doi: 10.1186/s13104-018-3866-8 (PMC6199704; doi:10.1186/s13104-018-3866-8)

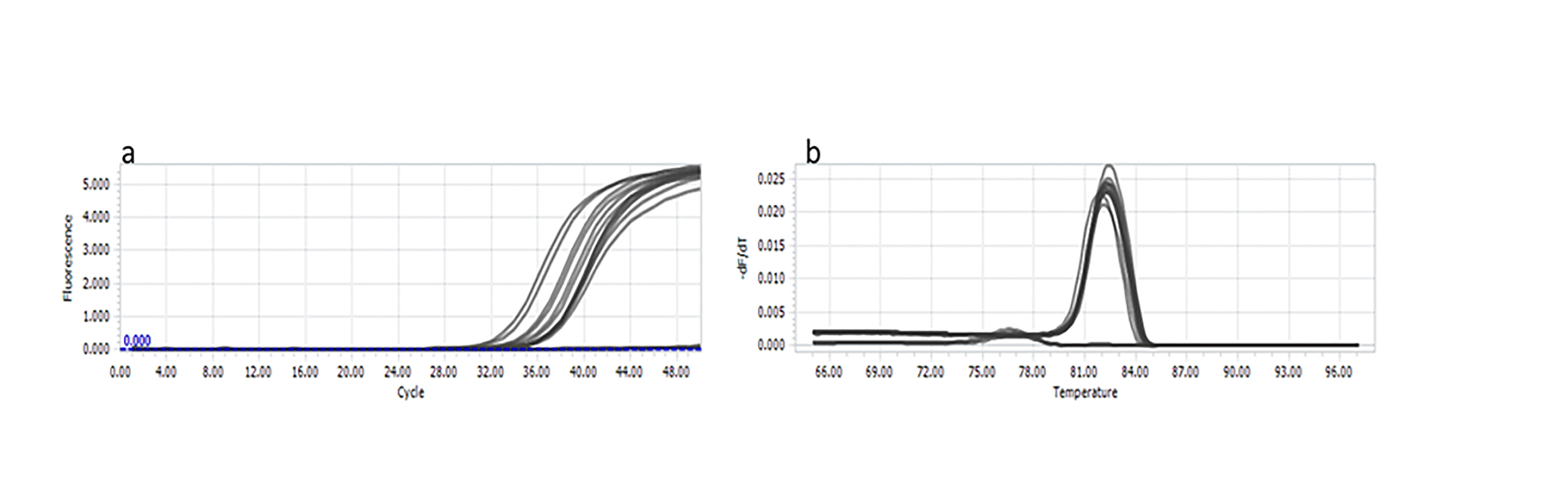

Supplement: Supplementary file 2 — Additional file 2: Fig. S1. SYBR Green real-time PCR for DYS221 locus in male bearing pregnancy. (a) Amplification Curve. (b) Melting peak. [file 13104_2018_3866_MOESM2_ESM.tif]

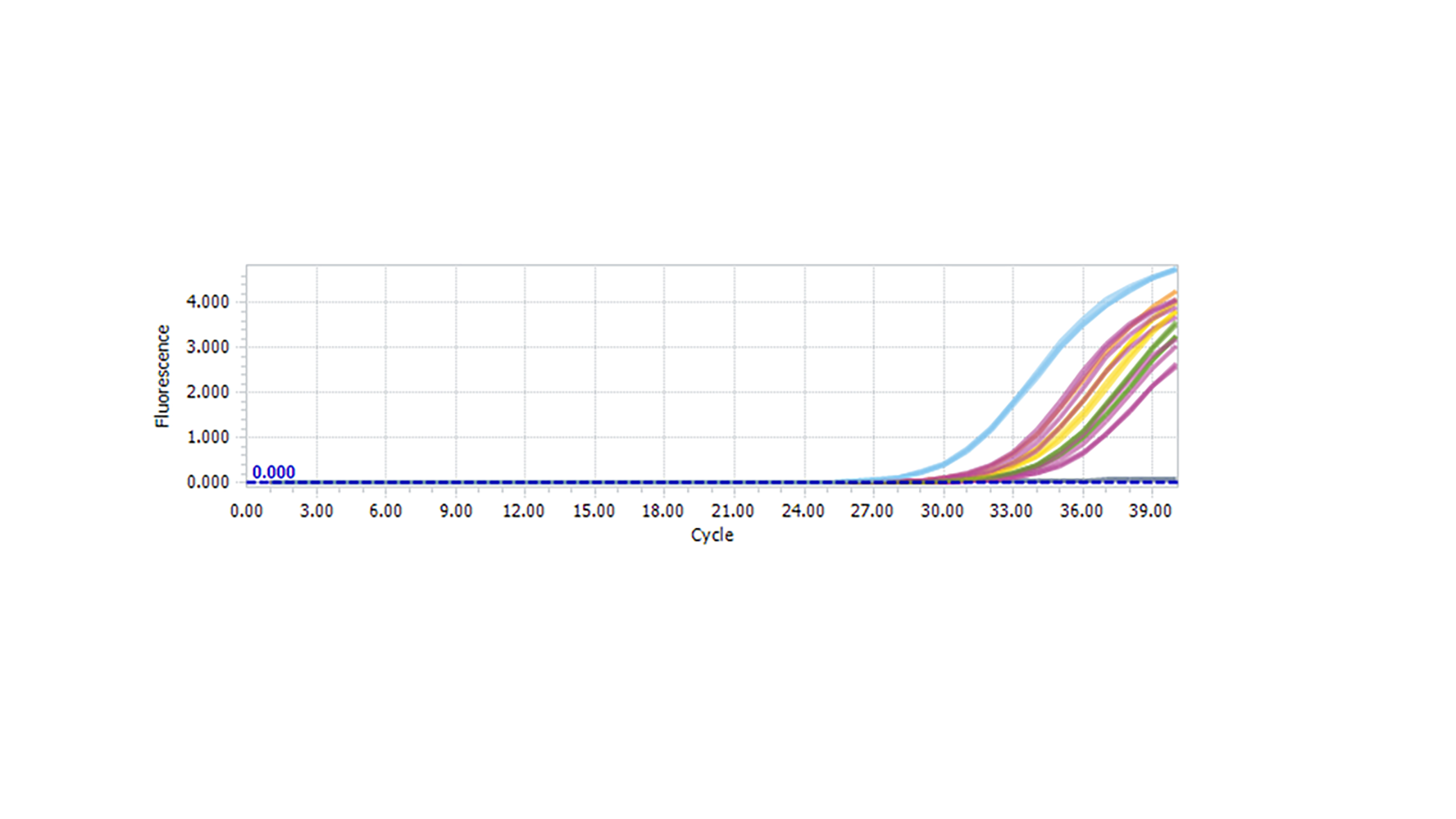

Supplement: Supplementary file 3 — Additional file 3: Fig. S2. Amplification curve of BMP3 gene in following spiked bisulfite DNA experiment. [file 13104_2018_3866_MOESM3_ESM.tif]

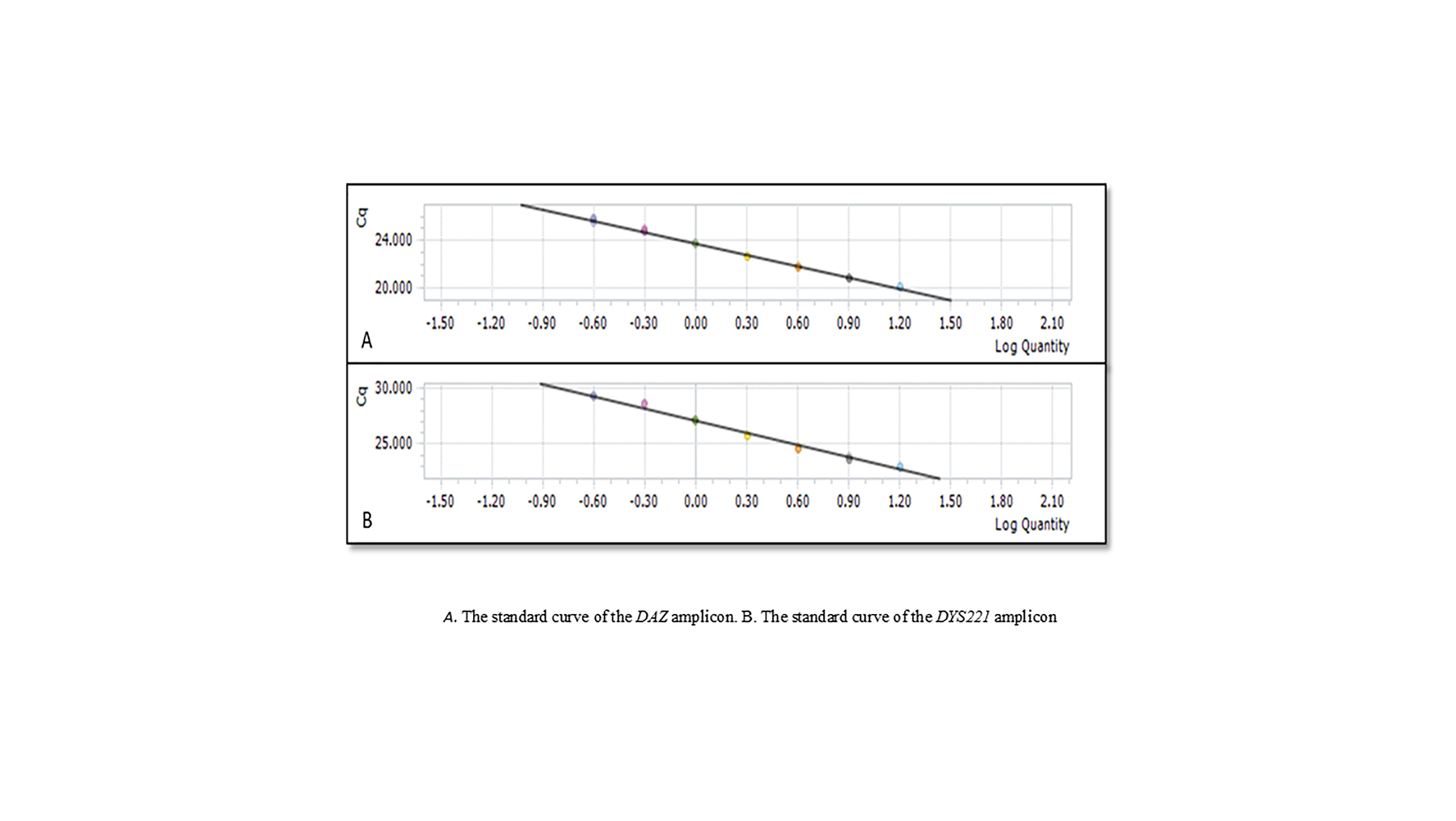

Supplement: Supplementary file 4 — Additional file 4: Fig. S3. The standard curve of the DAZ and DYS221 amplicon. [file 13104_2018_3866_MOESM4_ESM.tif]
